# Tool Wear Mechanism in Cutting of Stack CFRP/UNS A97075

**DOI:** 10.3390/ma11081276

**Published:** 2018-07-25

**Authors:** Severo Raul Fernandez-Vidal, Sergio Fernandez-Vidal, Moises Batista, Jorge Salguero

**Affiliations:** Industrial Design Department, Faculty of Engineering, Mechanical Engineering, University of Cadiz, Av. Universidad de Cádiz 10, E-11519 Puerto Real-Cádiz, Spain; sergio.fernandezvidal@mail.uca.es (S.F.-V.); moises.batista@uca.es (M.B.); jorge.salguero@uca.es (J.S.)

**Keywords:** wear, drilling, machining, dry drilling, stack, FML, CFRP, UNS A97075

## Abstract

The aeronautics industry’s competitiveness has led to the need to increase productivity with one shot drilling (OSD) systems capable of drilling stacks of dissimilar materials (fibre/metal laminates, FML) in order to reduce riveting times. Among the materials that constitute the current aeronautical models, composite materials and aluminium (Al) and titanium (Ti) alloys stand out. These one-pass machining techniques produce high-quality holes, especially when all the elements that have to be joined are made of the same material. This work has followed a conventional OSD strategy and the same cutting conditions applied to CFRP (carbo-fibre-reinforced polymer), Al and CFRP/Al stacked sheets to know the wear mechanisms produced. With this purpose, results were obtained by using current specific techniques, such as microstructural analysis, monitoring of the shear forces and analysis of macrogeometric deviations. It has been determined that when these drilling techniques are applied under the same cutting conditions to stacks of materials of a different nature, the results of the wear mechanisms acting on the tool differ from those obtained when machining each material separately. This article presents a comparison between the effects of tool wear during dry drilling of CFRP and UNS A97075 plates separately and when machined as stacks.

## 1. Introduction

The aeronautical sector has always been a benchmark in research, development and innovation. This has been motivated by the intense competitiveness that exists within the sector, generating a continuous need to improve functional, environmental and energetic efficiency in the processes, guaranteeing quality and seeking a direct impact on economic performance [1].

The first challenge the aerospace industry faces in its fourth revolution is to automate processes that nowadays include the extensive use of manual labour, especially in relevant operations such as assembly operations [2].

Among the different joining methods available in the industry, riveting is still most often used, regardless of the materials involved in the assembly. This joining process requires a previous drilling operation. OSD techniques can produce high-quality holes, especially in cases where all the elements to be joined are made of the same material [3,4].

In the construction of the latest aircraft models, the excellent characteristics of the carbon-fibre-reinforced thermoset matrix composites (CFRP) have formed a balance with the current light metal alloys (aluminium alloys 2XXX and 7XXX and titanium alloys Ti6Al4V) [3,4,5,6]. These structures are known as fibre/metal laminates (FML). The present study focuses on comparisons when facing drilling stacks of diverse types of material, resulting in a very different situation from machining them separately [7].

This case requires us to study the wear mechanisms that affect the tool during the drilling process of composite material stacks with metal alloys, relating them to the final quality of the drill in order to improve the performance of the process. Although some authors use optical techniques to quantify wear, these techniques do not offer a continuous record of wear and tear, and are difficult to integrate into the company’s intelligent systems. It has become necessary to monitor continuous variables directly related to tool wear during drilling, such as axial force [7]. This will favour the virtual integration of the company, being one of the main paradigms pursued in industry 4.0. The techniques used to carry out the analysis include microstructural analysis, monitoring of the shear forces and analysis of macrogeometric deviations.

### 1.1. Tool Wear Mechanisms in CFRP Drilling

**Figure 1:**
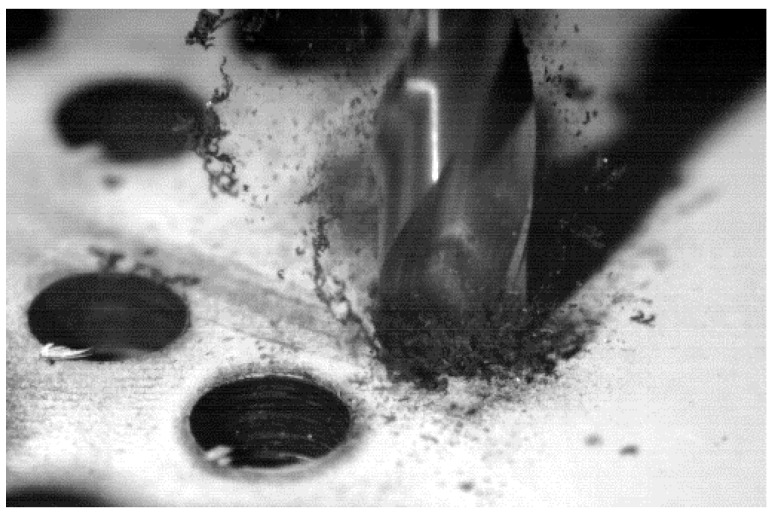
Evacuation of the composite material by dry OSD.

The conventional drilling process of CFRP involves continuous interaction between the tool and two types of materials. One is more plastic (matrix) and easily meltable at low temperatures, which can cause its thermal fixation to the tool. The other one, of higher hardness, is discontinuous in the matrix such as carbon fibre (CF); it is easily breakable and its particles (interacting with the edge, the release face and the evacuation channel of the drill bit) act in an abrasive way (Figure 1).

The abrasive action occurs in two different ways:

1. Direct. The particles of CF impact at high speed on the tool, producing a microblasting effect that reduces the cutting angle and the rigidity of the tool. When this action is combined with the cutting effect at the closest areas to the edge, dents can occur in the tool material, resulting in chipping or in a loss of sharpness of the tool material, also known as rounding [8,9,10].
2. Indirect. Once impacted over the tool, the dragged material causes its own removal in the direction of evacuation, which may develop longitudinal traces that accumulate tensile stresses. These reduce the tool resistance to compression and may cause its premature failure [11,12,13].

### 1.2. Tool Wear Mechanisms in Aluminium Drilling

The main wear mechanism during the machining of aluminium alloys is adhesion. When the material that has to be machined comes into contact with the tool surface, it creates bonding forces much stronger than the mechanical strength of the materials in contact, resulting in the transfer of particles from one surface to another [14]. This type of wear can occur in two ways:

1. Primary or direct adhesion. The particles of the tool are adhered to the chip being welded by the action of the forces developed in the tool–material interchange. In this case, when the yield stress of the chip is higher than the breakage limit of the adhered particles, these are pulled out of the tool and transported by the chip. This event can also promote abrasion on the release side due to the friction caused by these particles [15,16,17].
2. Secondary or indirect adhesion. This occurs when the machined material is incorporated into the tool, modifying its initial conditions. Depending on where it is located in the cutting tool, it is called [17]:
  - Adhesion on the cutting edge or raised edge (BUE—built-up edge).
  - Adhesion on the release side (BUL—built-up layer).

This phenomenon involves modifications in the properties of the cutting edge, with successive layers of machined, welded and hardened material becoming part of the edge. As mentioned before, wear is a dynamic mechanism, so the filler edge or BUE can be detached and regenerated, slowly removing material from the tool and causing primary or direct adhesion [17].

The friction of the chip with the tool within a certain temperature range increases the affinity between the materials of the tool and the piece, which promotes wear by adhesion. This situation is usually mitigated by high cutting temperatures [16].

**Figure 2:**
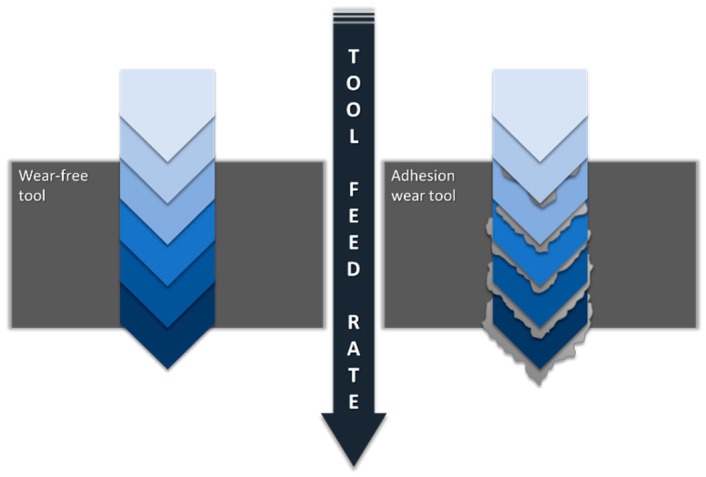
Influence of adhesive wear on the surface, dimensional and geometric quality of the borehole.

Continuous action of primary and secondary adhesion causes premature wear of the cutting tool, causing geometrical variations that affect both surface finishing and dimensional and geometrical tolerances (Figure 2).

## 2. Materials and Methods

### 2.1. Materials

**Figure 3:**
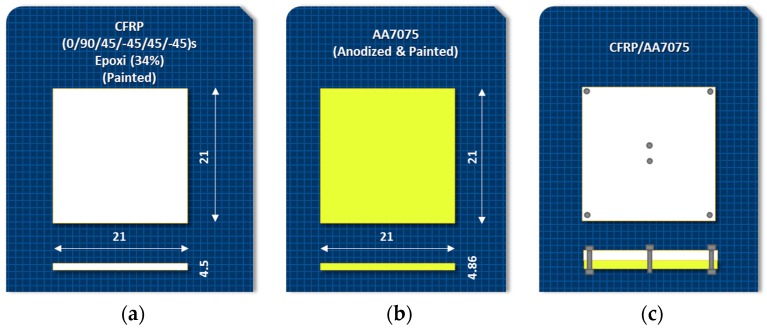
Characteristics and configuration of test materials: (**a**) CFRP; (**b**) UNS A97075 and (**c**) stacked CFRP/UNS A97075.

The materials used were presented in 210 × 210 mm^2^ sheets with thicknesses of 4.5 mm for CFRP and 4.86 mm for UNS A97075-T6 (UNS A97075). Figure 3 shows the different configurations. These materials have been selected for their importance in commercial airships.

The CFRP composite was made using unidirectional prepregs supplied by Hexcel Composite Company (Stamford, Connecticut, United States) referenced under HEXPLY M21/34%/194/T800S-24K (resin/resin content by weight (%)/fibre weight (gsm)/fibre type). The mechanical properties of the prepregs are density 1.28 g/cc, flexural yield strength 147 MPa, flexural modulus 3.50 GPa, flexural strain at yield 5%, glass transition temperature 185 °C. The lay-up sequence of the CFRP was (0/90/45/-45/45/-45)S so as to get a quasi-isotropic laminate. Aluminium alloy UNS A97075-T6 (composition Al 88.78%, Cu 1.87%, Mg 2.62%, Mn 0.08%, Zn 6.03%, Ti 0.11%, other 0.02%) was used on the other sheet. The properties of the aluminium alloy are: density 2.81 g/cc, ultimate tensile strength 524 MPa, yield tensile strength 462 MPa, elongation at break 11%, modulus of elasticity 71.7 GPa.

### 2.2. Tools

**Figure 4:**
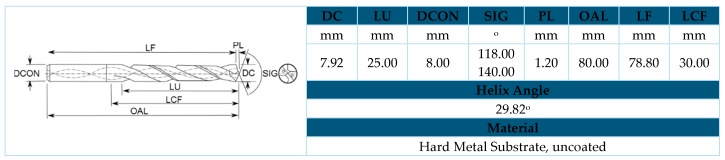
Characteristics of the drill bit used.

The chosen tool was a helical model from WC-Co without coating, being selected considering the materials that compose the stacks to be drilled, their thickness, the required qualities and cutting conditions. It has a double-angled tip; the section closest to the centre corresponds to the largest tip angle (140°) and the projection of the outer edges provides a tighter angle (118°) (Figure 4).

### 2.3. Equipment Used for the Operation and Evaluation of Drills

The selected OSD strategy has been the conventional dry type, applied separately to the CFRP and Al plank and the CFRP/Al stack. The stack configuration was CFRP/Al as it is the established machining sequence in the aerospace industry. This sequence aims to minimize the defects produced in the internal faces during the application of one-way assembly (OWA) techniques. The use of lubrication has not been considered, as we are looking to develop an environmentally friendly drilling process. The joining method has been defined in order to avoid possible failures caused by displacements of the plates that compose the stack.

The set values for the cutting parameters have been defined on the basis of other studies and real application cases, and are indicated in Table 1. Two tests were carried out using a Kondia Five 400 5-axis machining centre (Elgoibar, Guipuzcoa, Spain), controlled by a Heidenhain iTNC530 control system (Traunreut, Bavaria, Germany).

Microstructural analysis has been developed using scanning electron microscopy (SEM,) techniques. The compositional analysis of the materials and the cutting tool were carried out by means of EDS (energy-dispersive spectrometry) techniques with analytical capacity. The equipment used for the application of SEM and EDS techniques was the EDAX EDS System (Mahwah, NJ, USA).

For monitoring the shear forces, a dynamometer table model KITSLER© 9255B (Fx, Fy, Fz and Mz) (Kistler Holding AG, Winterthur, Zürich, Switzerland) was used. This instrument is connected to a computer that transfers the obtained data to the computer using Labview software (National Instruments, 2014, Austin, TX, USA) for processing. The sampling rate is 1000 Hz.

One of the analysis parameters studied within the macrogeometric deviations was the dimensional tolerance of the hole diameters. This measurement was carried out with a Mitutoyo three-contact internal micrometre (Mitutoyo Corporation, Kawasaki-shi, Kanagawa, Japan) with a measuring range of 6–8 mm, an accuracy of 0.001 mm and a measurement uncertainty of 2 µm. A total of three were measured sizes at different heights and angles per hole in each material.

## 3. Results and Discussion

### 3.1. Tool Wear during CFRP Drilling

**Figure 5:**
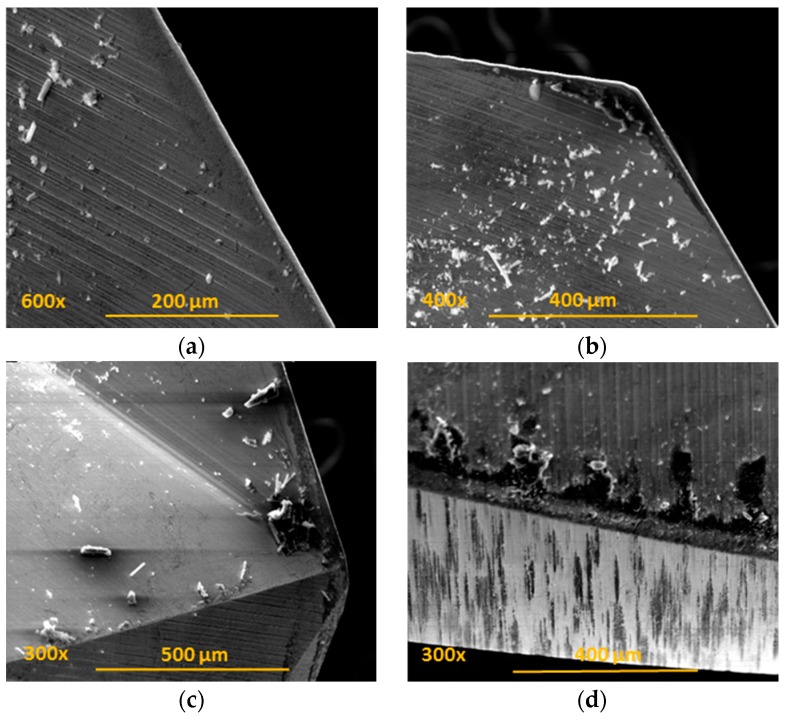
Location of drill wear by the abrasive action of carbon fibres: (**a**) main edge; (**b**) primary and secondary edge union; (**c**) tool tip; (**d**) peripheral face.

Through the observation of different images obtained by SEM, abrasion was ratified as the main wear mechanism as a consequence of the continuous cutting action of carbon fibre (CF) [11,12,13]. Its geometric effects along the tool were visible when the tool surface’s own marks disappear (Figure 5a). Other consequences found were erosion of the cutting edges (Figure 5b) and the tool tip (Figure 5c), and the appearance of marks along the periphery of the drill bit (Figure 5d).

**Figure 6:**
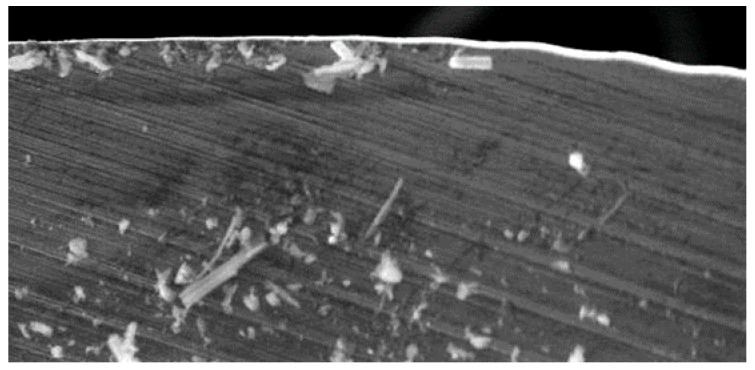
Detail of irregularities in the cutting edge of the tool.

The continuous action of the carbon fibre on the cutting edge of the tool caused its irregularity as a consequence of abrasion [8,9,10] (Figure 6).

**Figure 7:**
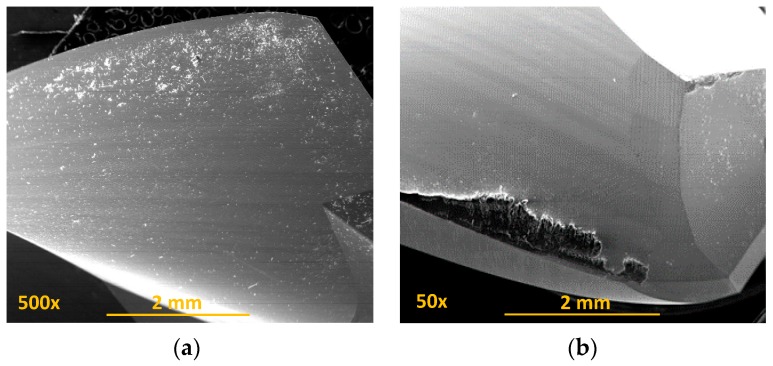
Accumulation of carbon fibre adhered to the tool: (**a**) face rake; (**b**) the area next to the guide surface of the tool.

The incipient fusion of the epoxy matrix may cause its dispersed adhesion over the tool. Adhered elements in the tool can incorporate particles or small pieces of CF that interfere with machining, especially when located in chip evacuation channels. A considerable amount of adhered material (Figure 7) was observed, which could be a signal of further deterioration of the matrix in the walls of the drilled material. The absence of resin is the cause of the problematic loss of the fibres junction, with a consequent decrease in the quality of the hole.

### 3.2. Tool Wear in Conventional Drilling UNS A97075

**Figure 8:**
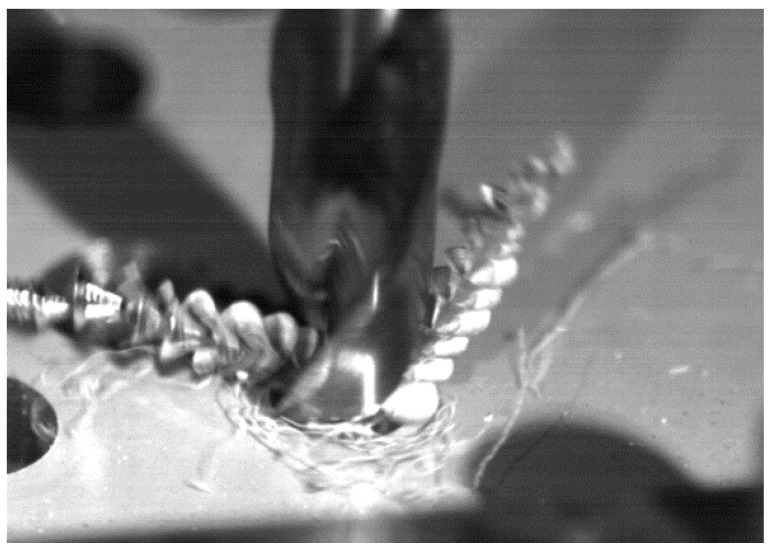
Chip evacuation during dry drilling of alloy UNS A97075.

During conventional drilling of the aluminium alloy UNS A97075, the interaction between the workpiece and the tool caused a large helical chip (Figure 8), which makes machining difficult [2]. The zinc (Zn) contained in the UNS A97075 alloy provides a high degree of plasticity, making breakage difficult due to its ductility [3].

**Figure 9:**
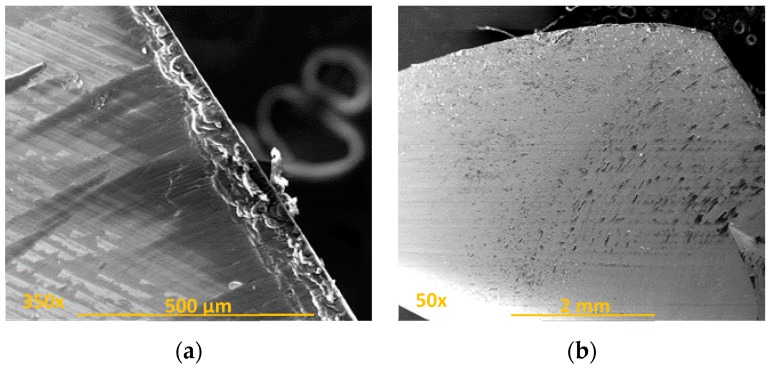
Wear by bonding mechanisms (BUL–BUE) on the tool after conventional drilling of the aluminium alloy UNS A97075: (**a**) Main cutting edge; (**b**) evacuation channel.

The secondary adhesion of the aluminium alloy on the tool was the main wear mechanism detected. This was favoured by the absence of refrigeration and lubrication, as happened in this case. Thus, in the first stage, the aluminium matrix was melted down and welded onto the tool surface, forming a pure Al layer and giving rise to the primary BUL (Figure 9a). This circumstance provided the conditions for the mechanical adhesion of the alloy, mainly in the areas of the edge, resulting in a built-up edge (BUE) that grew to a critical size at which it began to extrude, giving rise to a secondary BUL that was deposited as a second layer over the primary BUL [4,5,6,17].

The location of the BUE is usually over boundary edge zones, but the BUL, during the drilling, may extend to areas outside the rake face, such as the chip evacuation channel, seriously hampering the quality of the process (Figure 9b).

**Figure 10:**
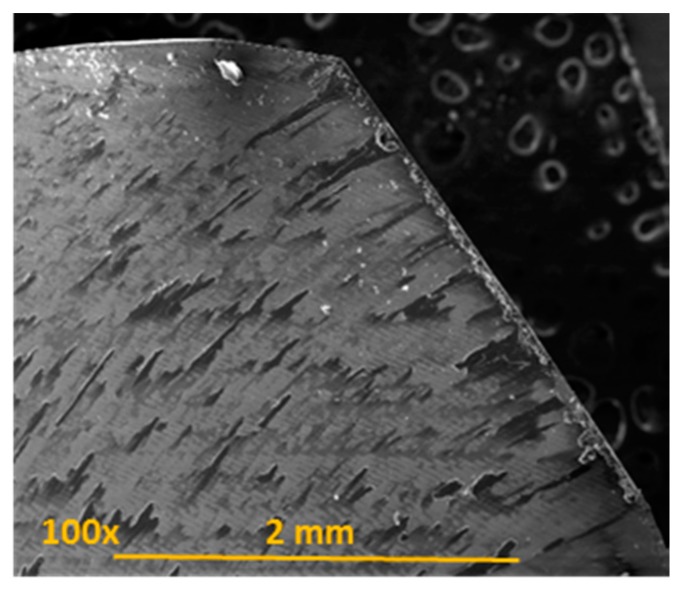
Increased bond wear with the number of holes.

The raised friction in the piece–tool–chip interactions during cutting resulted in an elevation of the process temperature, increasing the affinity between the tool material and the aluminium, and intensifying the plasticity of the alloy, which facilitated this type of wear [7,8,9]. This process caused higher adhesion of the aluminium over the rake face, as in the regrowth of the cutting edges (Figure 10).

**Figure 11:**
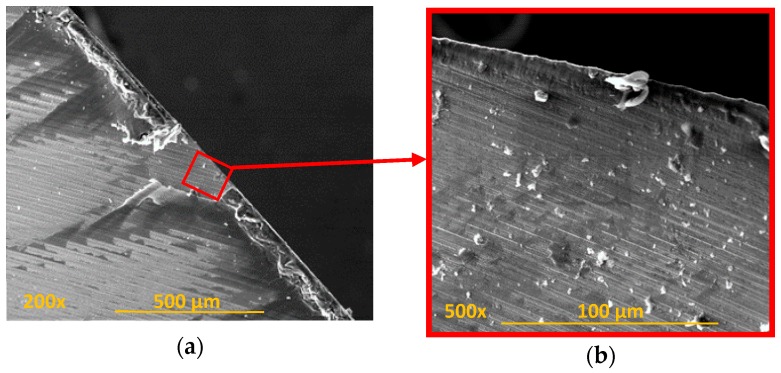
(**a**) Primary adhesion mechanism; (**b**) geometric irregularities observed in the cutting edges.

The analysis of the cutting edges showed irregularities. These were produced by the dynamic phenomenon of primary adhesion, which, with the detachment of the BUE and its new generation, slowly removes material from the tool (Figure 11a). At the same time, this action can cause abrasive wear on the tool surface or affect the quality of the surface generated on the workpiece itself (Figure 11b).

### 3.3. Tool Wear in Conventional Stack Drilling CFRP/UNS A97075

When drilling stacks of CFRP/UNS A97075, the drill bit comes into contact with two materials at the same time and acts on them with similar parameters.

**Figure 12:**
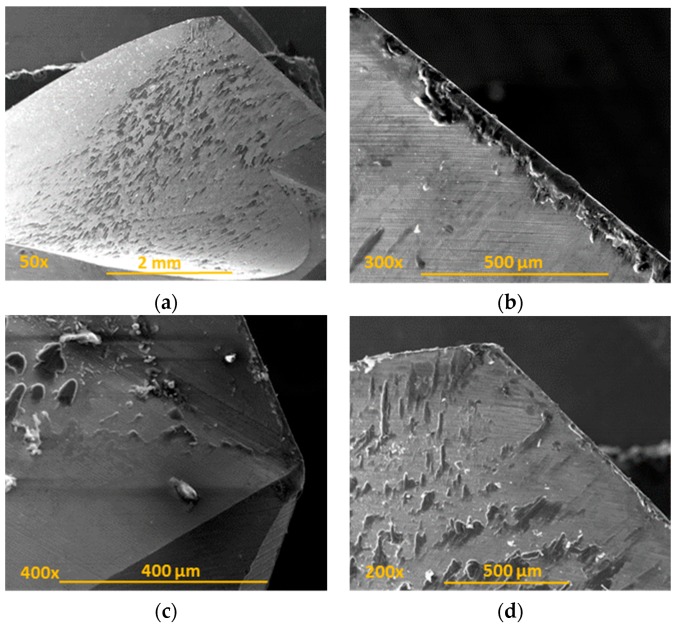
Wear located on the tool after conventional drilling of the CFRP/UNS A97075 stack: (**a**) Flute (BUL); (**b**) cutting lip (BUE y BUL); (**c**) tool tip; (**d**) joining of primary and secondary cutting edges (BUL).

Observation of the tools showed how adhesion was the main type of wear in the conventional drilling of this stack. During machining, the rake face was subjected to increased pressure and temperature [18,19], hence helping in the formation of an adhesive layer in the contact zone between the tool and the workpiece [14,20]. Adhesion wear was caused by the mechanical removal of the tool material when the adhesive junctions were broken. The effects of the abrasion wear mechanism were attenuated by the adhered material (Figure 12).

**Figure 13:**
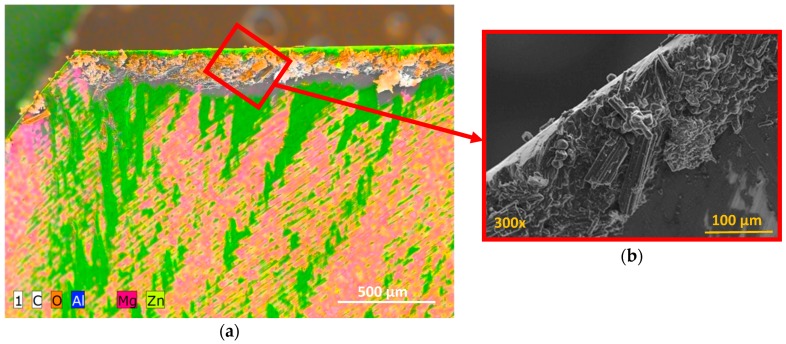
(**a**) Composition analysis of the BUE and release side after dry drilling of the stack CFRP/UNS A97075; (**b**) details of the BUE with carbon nanotube adhered to aluminium.

It was seen how the adhered material was composed of both aluminium alloy and carbon fibre particles. This caused a geometric change in the tool, affecting the final quality of the hole [20,21,22] but also its mechanical characteristics, since the incorporation of the Al–CFRP mixture was abrasive to the material that had to be machined (Figure 13).

**Figure 14:**
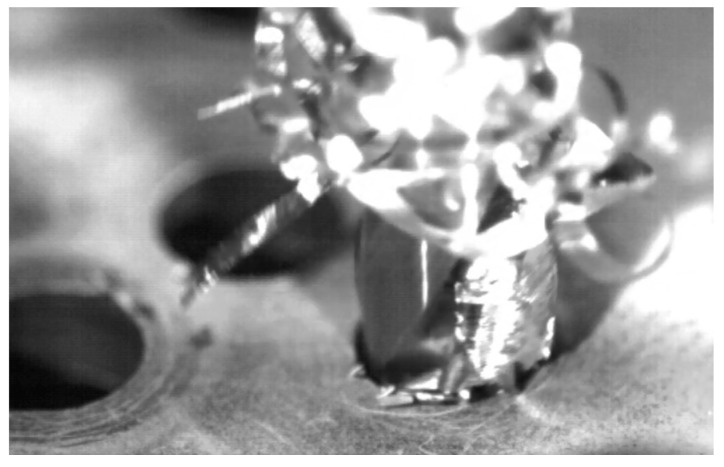
Chip removal during stacking drilling CFRP/UNS A97075 using conventional technology.

This blend of aluminium and CF seemed to facilitate chip adhesion along the tool during dry drilling of CFRP/ UNS A97075, as shown in Figure 14.

One method to decrease wear during the drilling process is to reduce the temperature. This can be done by the use of lower cutting speeds and higher feed rates and the use of advanced techniques (vibration-assisted drilling, strategies with minimum quantity lubricant (MQL) and cryogenic machining, for instance).

### 3.4. Driven Force in CFRP, UNS A97075 and CFRP/UNS A97075 Stacks

**Figure 15:**
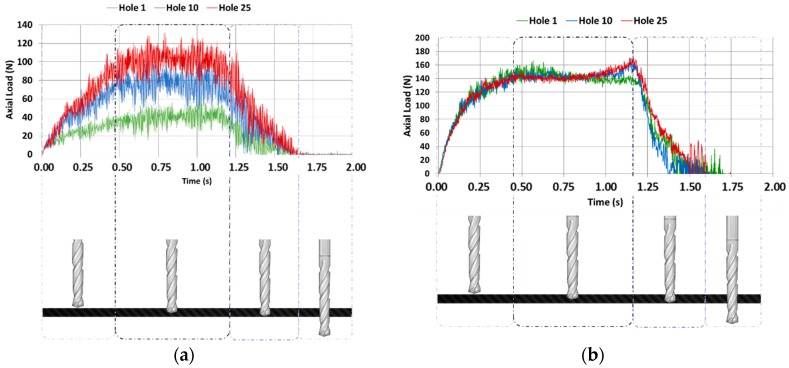
(**a**) Axial force with respect to the time applied to the drilling of CFRP plate; (**b**) axial force with respect to the time applied to the drilling of UNS A97075 plate.

Figure 15a shows the values of the thrust force with regard to the height of the tool in CFRP plate, while Figure 15b shows the results obtained in UNS A97075 plate. In the first case (CFRP), the force when the tool is fully embedded in the material was increased by the number of holes drilled, from approximately 40 N to 100 N. This might be due to the rounding of the cutting edge by the abrasive action of the fibre. The force level remained significantly lower than the aluminium alloy with the same number of holes drilled. In this second case, the force remains almost constant after 25 holes. Adhesion wear does not appear to be significant.

**Figure 16:**
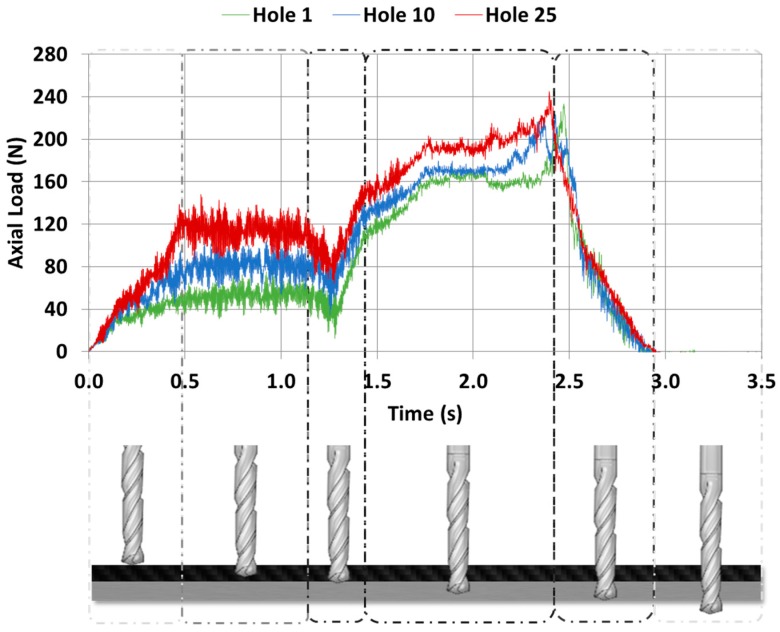
Axial force respecting the time applied to the drilling of CFRP/UNS A97075 stack.

Evolution of the values of thrust forces regarding to the feed rate of the tool in the stack is shown in Figure 16. The axial load values were increased in both materials with the number of holes.

A potential model was used to represent tool wear, as suggested by different studies [4,7,23]:

(1) $$Fz=KN^{m},$$

where *Fz* is the axial force, *K* is a constant depending on the geometry of the tool and the properties of the material being machined, *N* is the number of holes, and *m* measures the degree of wear of the tool.

**Figure 17:**
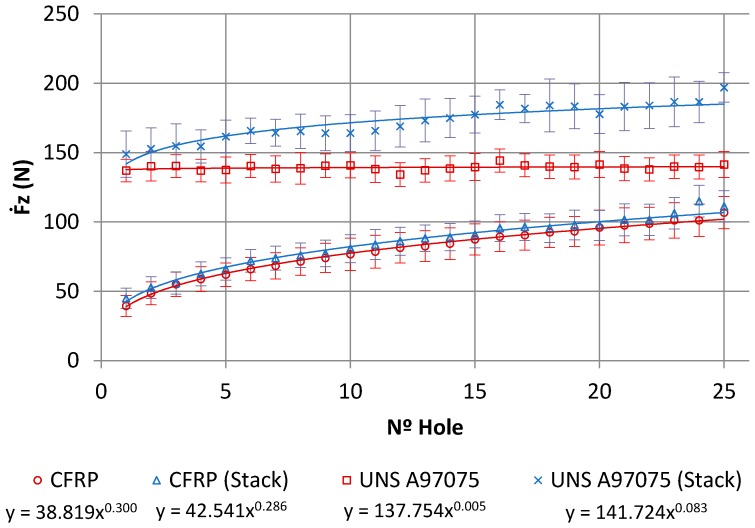
Axial load versus number of holes for CFRP and Al alloy plates alone and CFRP/UNS A97075 stack.

The axial force values and their models for the same number of holes are shown in Figure 17. The mean axial drilling forces in CFRP separately go from 39.44 N to 106.72 N, with deviations from 7.5 N to 12.78 N. The mean axial drilling forces in Al separately go from 136.96 N to 141.44 N, with deviations from 8.12 N to 11.36 N. The mean axial drilling forces in CFRP in stack go from 44.94 N to 111.15 N, with deviations from 7.26 N to 11.44 N. The mean axial drilling forces in Al stack drilling range from 148.95 N to 197 N, with deviations from 7.2 N to 11.59 N. The force values during stack machining were similar to those for CFRP plank separately, but were highly increased by the number of holes in comparison to the Al plank. This might be caused by the increased friction due to the adhesion of Al and CF, which resulted in an elevation of the process temperature. This increased the affinity between the tool material and the aluminium and intensified the plasticity of the alloy, favouring the generation of BUE [7,8,9]. The wear of the drill bit seems to be more influenced by the adhesion wear of the Al than by the abrasion of the CFRP.

### 3.5. Hole Quality in Drilling: Diameter

**Figure 18:**
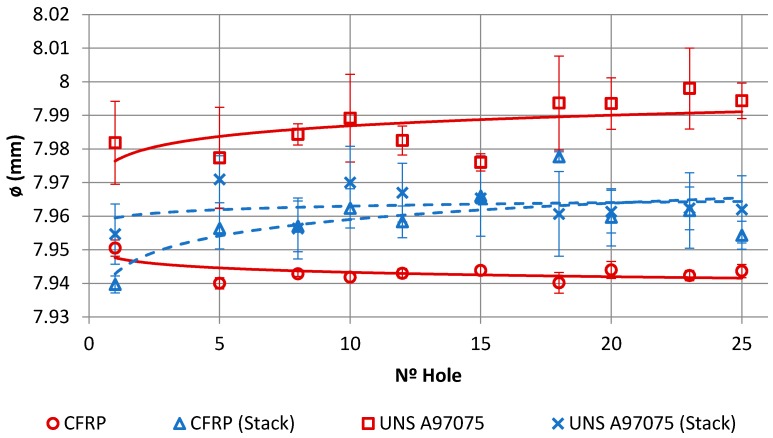
Evolution of hole diameter with number of holes.

Figure 18 shows the evolution of the hole diameter with the number of holes measured on both materials when machined separately or in a stack. The mean diameters in the separate CFRP drilling range from 7.95 to 7.94 mm, with deviations from 0.001 to 0.002 mm N. The mean diameters in the separate Al drilling range from 7.98 to 7.99 mm, with deviations from 0.002 to 0.014 mm. The average diameters in the drilling of CFRP in stack go from 7.93 to 7.97 mm, with deviations from 0.001 to 0.003 mm. The average diameters in the Al stack drilling range from 7.95 to 7.97 mm, with deviations from 0.001 to 0.012 mm. There were discrepancies in the diameter between the CFRP and UNS A97075 plates, with the lowest and most stable values being the CFRP ones. The general trend of CFRP drilling is that the diameter decreases with the number of holes, which can be attributed to the progressive loss of material in the tool due to the abrasion effect of CF, while the diameter of the hole in the plate of UNS A97075 is the opposite, attributed to the adhesion effect of the cut material produced.

When machining the stack, there was a variation in the diameter of the holes compared to machining the same material separately. The CFRP inlet could cause a deterioration in the hole in this material in the evacuation of Al that can be attributed mainly to the abrasion on the surface of the hole caused by the rotation of the aluminium chip next to the drill bit. On the contrary, in UNS A97075 the variation in the diameter of the holes was reduced, possibly as a result of the negative synergies produced by the CFRP and the UNS A97075 on the tool. The discrepancy between the diameters could also be related to the different temperatures developed during drilling. As a result, thermal deformations in the planks may vary and lead to different diameters.

## 4. Conclusions

This paper reports a comparison of the wear mechanisms produced during the dry drilling of CFRP and UNS A97075 planks separately and when machined as stacks. 

Tool wear combines the effects of CFRP and UNS A97075 alloy machining. In CFRP drilling, the fundamental wear is the abrasion by impact and/or drag of the carbon fibre particles removed during the process. However, adhesion is detected by incipient fusion of the epoxy matrix, which in turn incorporates CF particles. This makes chip evacuation difficult, especially when combined with drilling of the metal alloy.

In the drilling of alloy UNS A97075, the wear mechanism includes different stages. The secondary adhesion of the aluminium alloy on the tool is the main wear mechanism detected. In the first stage, the aluminium matrix is melted and welded onto the tool surface, forming a pure Al layer. This creates the primary BUL, which facilitates the mechanical adhesion of the alloy, mainly in the areas of the edge and making up a BUE that grows to a critical size, from which it begins to extrude. This leads to a secondary BUL, deposited as a second layer over the primary BUL.

The interaction of CFRP and Al materials when drilled together is reflected by the comparison of wear when drilled separately. Bond wear seems to predominate over abrasion of the tool surface. The BUE generated on the edge of the tool contains CF fragments that seem to facilitate the adhesion of aluminium and reduce the diameter differences in the drilling of materials.

## Figures and Tables

**Table 1:** Tested cutting conditions.

Diameter (mm)	Cutting Speed (m/min)	Feed Speed (mm/min)	Holes (n)	Lubrication
7.92	145	250	25	Dry
